# Timing is everything – obtaining accurate measures of plant uptake of amino acids

**DOI:** 10.1111/nph.17964

**Published:** 2022-02-02

**Authors:** Henrik Svennerstam, Sandra Jämtgård

**Affiliations:** ^1^ Umeå Plant Science Centre Department of Forest Genetics and Plant Physiology Swedish University of Agricultural Sciences Umeå SE‐901 83 Sweden; ^2^ Forestry Research Institute of Sweden (Skogforsk) Box 3 Sävar SE‐918 21 Sweden; ^3^ Department of Forest Ecology and Management Swedish University of Agricultural Sciences Umeå SE‐901 83 Sweden

**Keywords:** amino acids, *Arabidopsis thaliana*, EA‐IRMS, glutamine, isotopes, LC‐MS, organic nitrogen, plant uptake

## Abstract

- Plants are known to have the capacity to take up and utilise amino acids for growth. The significance of this uptake, however, remains elusive, partly due to methodological challenges and biological implications associated with acquiring and interpreting data.
- This study compared bulk stable isotope analysis and compound‐specific liquid chromatography‐mass spectrometry, two established methods for determining amino acid uptake. Root amino acid uptake was assayed using U‐^13^C_5_‐^15^N_2_‐l‐glutamine and axenically grown *Arabidopsis thaliana*.
- After 15–120 min of exposure, the content of intact glutamine measured in the roots was constant, whilst the ^15^N and ^13^C content increased over time, resulting in very different estimated uptake rates. The ^13^C : ^15^N ratio in roots declined with time, suggesting a loss of glutamine carbon of up to 15% within 120 min.
- The results presented indicate that, regardless of method used, time is a crucial factor when determining plant amino acid uptake. Due to post‐uptake metabolism, compound‐specific methods should primarily be used in experiments with a time frame of minutes rather than hours or days. Post‐uptake metabolism in plants may account for significant loss of carbon, suggesting that it is not just pre‐uptake metabolism by microbes that accounts for the ^15^N–^13^C mismatch reported in ecological studies, but also post‐uptake metabolism in the plant.

## Introduction

The capacity of plant roots to take up amino acids (AAs) has been known for some time now and is widespread among plant species (Hutchinson & Miller, 1912; Chapin *et al*., 1993; Näsholm *et al*., 2009). Despite this and the fact that the mechanisms responsible for root uptake of AAs have been identified (Rentsch *et al*., 2007; Tegeder & Rentsch, 2010), the quantitative importance of AAs in terms of plant nitrogen nutrition is still debated (Jones *et al*., 2005; Kuzyakov & Xu, 2013). The reason for this is the transient nature of AAs, mainly as a consequence of (1) pre‐uptake soil microbial cleavage, (2) the occurrence and rate of replenishment of AAs in soil (although outside the scope of the present study) and (3) post‐uptake plant metabolism. These three obstacles make quantification of AA uptake in plants challenging (Sauheitl *et al*., 2009; Warren, 2012; Enggrob *et al*., 2019; Hill & Jones, 2019).

The most frequently used method for analysis of AA uptake involves the use of dual‐labelled (^13^C, ^15^N) AAs and analysis by elemental analysis combined with isotope‐ratio mass spectrometry (EA‐IRMS), sometimes referred to as bulk stable isotope analysis (BSIA). The method has, however, been criticised for its potential overestimation of intact uptake due to the uptake of labelled compounds that are the product of microbial decomposition (Rasmussen & Kuzyakov, 2009; Sauheitl *et al*., 2009).

Because of these issues and recent refinement of analytical methods, there has been a shift towards compound‐specific analysis of isotopic tracers to quantify AA uptake (Sauheitl *et al*., 2009; Dippold & Kuzyakov, 2013; Moran‐Zuloaga *et al*., 2015). There are two main types of methods: compound‐specific stable isotope analysis (CSIA) (Sauheitl *et al*., 2009; Dion *et al*., 2018) and analysis of the intact isotopically labelled AA (Warren, 2012; Czaban *et al*., 2016; Ganeteg *et al*., 2017). The two techniques differ in that CSIA quantifies the amount of ^15^N and ^13^C in each AA pool, whilst analysis of the intact isotopically labelled AA quantifies the AA isotopologues, molecules that differ in their isotopic composition, in which one molecule has at least one atom with a different number of neutrons than the parent molecule. The main conclusion from many of the studies using these techniques is that BSIA overestimates AA uptake (Sauheitl *et al*., 2009; Czaban *et al*., 2016; Dion *et al*., 2018) due to soil microbial activity before plant uptake.

It has often been argued that plants are inferior competitors for organic nitrogen compared with microbes (Hodge *et al*., 2000; Jones *et al*., 2005; Kuzyakov & Xu, 2013) and that the de‐coupling of ^13^C and ^15^N following microbial decomposition of dual‐labelled AAs has implications for the quantification of plant AA uptake. This is because microbial decomposition of AAs may cause the release of compounds carrying ^13^C and/or ^15^N labels, compounds that plants have the capacity to take up (Rasmussen & Kuzyakov, 2009; Sauheitl *et al*., 2009; Moran‐Zuloaga *et al*., 2015; Hill & Jones, 2019). It is therefore possible that these compounds may be released from the microbes and are then taken up by plants, either alone or together, leading to (1) the aforementioned overestimation of intact AA uptake or (2) a shift in the ^13^C : ^15^N ratio, making data interpretation challenging. An important issue that is often overlooked when making these methodological comparisons is the parallel activity of AA post‐uptake metabolism in plants (Warren, 2012). The simultaneous post‐uptake metabolism of AAs inside the plant may also shift the ^13^C : ^15^N ratio. In systems with both plants and microbes present it is therefore difficult to distinguish which organism is responsible for metabolising or decomposing any introduced AAs.

Using Arabidopsis mutant lines and overexpressors with low and high AA transporter expression, ^15^N‐ and ^13^C dual‐labelled glutamine (Gln), BSIA and compound‐specific liquid chromatography‐mass spectrometry (LC‐MS), Ganeteg *et al*. (2017) were able to demonstrate a correlation between AA transporter expression and AA uptake in agricultural soil. Moreover, based on Ganeteg *et al*.'s (2017) data collected using BSIA and compound‐specific LC‐MS after 15 and 60 min of exposure, it is evident that the percentage recovery of ^13^C and ^15^N labels within intact Gln, compared with the amount measured using BSIA, decreased over time, suggesting that post‐uptake metabolism had occurred. However, even though clear differences were observed between the Arabidopsis lines used with respect to the uptake of intact AA, microbial decomposition before uptake was not measured and may have influenced the BSIA data.

Therefore, time and the timing of data collection are important factors when assessing plant post‐uptake metabolism, especially in relation to microbial decomposition before plant uptake. Expected half‐lives of AAs in soil range from only a few minutes to a few hours (Rousk & Jones, 2010; Hill & Jones, 2019) and time is also critical in relation to plant AA metabolism (Persson & Näsholm, 2001; Warren, 2012; Ganeteg *et al*., 2017), a matter that needs more attention. In both cases, it is critical to know how long the AA molecule remains intact. An additional complicating factor when quantifying plant AA uptake is that the replenishment of AAs in soil in each and every moment may be of greater importance than the resistance of a single AA molecule over time (Enggrob *et al*., 2019; Hill & Jones, 2019).

Given the risk of overestimating uptake rates with BSIA, the method that, historically, has been used most frequently, and the potential underestimation associated with the compound‐specific isotope methods that are increasingly being used, we wanted to compare the two methods and evaluate the influence of time on quantification, in the absence of soil microbes, with only plant metabolism affecting break down.

To examine this issue, U‐^13^C_5_‐^15^N_2_‐l‐Gln was applied to roots of axenically grown *Arabidopsis thaliana* for 15–120 min. This allowed us to focus on the effect of time on post‐uptake metabolism without any microbial activity interfering with the process. To compare uptake of the intact molecule against that of ^15^N ^13^C, ^15^N and ^13^C were analysed with BSIA and U‐^13^C_5_‐^15^N_2_‐l‐Gln and other AA isotopologues, in the free AA pool in root tissue were quantified using compound‐specific LC‐MS‐qTOF.

## Materials and Methods

*Arabidopsis thaliana* Col‐0 seeds were sown onto vertical agar plates containing the equivalent of half‐strength Murashige & Skoog (½MS) medium, but with N added as 3 mM NO_3_^−^, 0.5% sucrose and 1% plant agar (Duchefa, Haarlem, the Netherlands). After sowing, plates were kept at 4°C for 48 h to ensure even germination. The plants were then grown in a long day 16 h : 8 h, light : dark regime at 20°C for 20 d.

### Labelling experiment

The labelling/uptake experiment was carried out on sucrose‐ and nutrient‐free 1% plant agar (Duchefa) plates. Each plate was filled with 70 ml agar. The agar was relatively hard, meaning roots grew on top of the agar rather than down into it, facilitating handling of the plants. After 20 d of growth on the vertical plates, five plants with agar free roots were transferred and placed on top of the agar on horizontally oriented sucrose‐ and nutrient‐free plates, 5 ml of labelling solution was pipetted onto each plate to create a thin film covering the entire surface of the agar plate. The labelling solution contained 1.5 mM l‐Gln (U‐^13^C_5_, U‐^15^N_2_, 98%) and the equivalent of N‐free ½MS medium, a pH of 5.8 was achieved by the addition of NaOH. Assuming that the labelling solution is absorbed by the agar medium, substrate concentration would correspond to 100 μM. To avoid contact between shoots and the labelling solution, a platform made from aluminium foil was created to shield the shoot. Labelling lasted 15, 30, 45, 60 or 120 min and afterwards roots were rinsed in 0.5 mM CaCl_2_ three times. Plants were then separated into shoot and root before being immediately frozen in liquid nitrogen (*n* = 5, with each replicate being a pool of roots from five individual plants). The plant material was then freeze dried for 48 h. The roots were weighed and chopped up to homogenise them before further analysis.

### Chemical analysis

#### LC‐MS‐qTOF analysis of AA isotopologues

Free AAs in root tissue were extracted from freeze‐dried root samples and analysed with an Agilent 6540 UHD Accurate Mass QTOF LC/MS using an electrospray ionisation (Dual AJS ESI) probe following the methods described by Czaban *et al*. (2016) and Ganeteg *et al*. (2017). The gradient elution buffers were A (H_2_O, 0.1% formic acid) and B (acetonitrile, 0.1% formic acid) and the column used for separation of AAs was a Phenomenex C18 column (2.1 mm × 100 mm, 1.7 μm). The samples were derivatised with a Waters AccQ‐Tag™ Ultra Derivatisation kit for AA analysis. In total, 19 AAs (Ala, Arg, Asn, Asp, γ‐aminobutyric acid (GABA), Gln, Glu, Gly, His, Ile, Leu, Lys, Met, Phe, Pro, Ser, Thr, Tyr, Val) and their isotopologues including U‐^13^C_5_‐^15^N_2_‐l‐Gln, were identified based on retention time and mass to charge ratio (*m*/*z*); the mass of the AA isotopologue with the AccQ‐Tag™ attached. The derivatised AAs were detected on a mass spectrometer equipped with a jet‐stream electrospray source operating in positive ion mode. The jet‐stream gas temperature was 300°C with a gas flow of 8 l min^−1^, a sheath gas temperature equal to 350°C and a flow rate of 11 l min^−1^, nebuliser pressure was set to 40 psi. The capillary voltage was set to 4 kV and the nozzle voltage to 0 V. Reference mass correction was based on the reference mass *m*/*z* 922.0097.

Amino acid concentrations were measured according to the ultra‐performance LC (UPLC)‐AccQ‐Tag method (UPLC amino acid analysis system solution, www.waters.com).

The isotopologue concentrations (µmol g^−1^ dry weight (DW)) were calculated in two steps. Amino acid excess of each AA isotopologue was calculated with the online application FluxFix (http://fluxfix.science/, Trefely *et al*., 2016). In this application, the subtracted natural abundance was calculated by generating a correction matrix based on the natural abundance isotopologue data from the nonlabelled samples (from time point zero), which is used to transform the peak area values into percentage mole enrichment for each isotopologue measured (Trefely *et al*., 2016). The natural abundance from the AccQ‐Tag™ derivative was indirectly subtracted in the calculations.

The concentration (μmol g^−1^ DW) of each isotopologue [AA^+^ *^n^*] at each time point was calculated as:

$$\text{Excess}\;{\text{AA}}^{+n}\times \left[\text{AA}\right]=\left[{\text{AA}}^{+n}\right]$$

[AA], represents the total concentration of the AA in the sample.

#### EA‐IRMS analysis of ^13^C and ^15^N

Bulk stable isotope analysis of the ^13^C and ^15^N contents of the root samples was determined using an elemental analyser (Flash EA 2000) connected to a continuous flow isotope‐ratio mass spectrometer (DeltaV)–EA‐IRMS, (Thermo Fisher Scientific, Bremen, Germany) at the SLU Stable Isotope Laboratory (SSIL).

### Data processing and statistical analysis

Processing of LC‐MS data was conducted using matlab R2011b (Mathworks, Natick, MA, USA) and an in‐house database developed by the Umeå Plant Science Centre (UPSC). Statistical analyses were carried out in Minitab 17.1.0 (Minitab Inc., State College, PA, USA).

## Results

### LC‐MS‐qTOF quantification of U‐^13^C_5_‐^15^N_2_‐l‐Gln

**Fig. 1:**
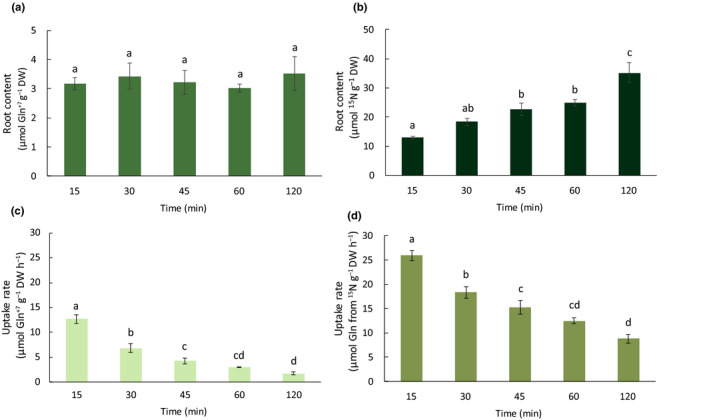
(a) Arabidopsis root content of Gln^+7^ after 15–120 min exposure to U‐^15^N_2_, ^13^C_5_‐Gln solution, determined by quadrupole time‐of‐flight liquid chromatography (LC‐qTOF). (b) Root content of ^15^N after 15–120 min exposure to U‐^15^N_2_, ^13^C_5_‐Gln solution, determined by bulk stable isotope analysis (BSIA). (c) Root uptake rates of Gln+7 determined by LC‐qTOF. (d) Root uptake rate of Gln calculated from ^15^N determined by BSIA. Mean ± SE, *n* = 5. Different lower case letters indicate significant differences between time points within each graph, tested with One‐way analysis of variance, Tukey's test, *P* ≤ 0.05.

Plant root uptake was determined by compound‐specific LC‐MS‐qTOF analysis on Arabidopsis roots exposed to U‐^13^C_5_‐^15^N_2_‐l‐Gln for 15–120 min. Intact free U‐^13^C_5_‐^15^N_2_‐l‐Gl n (from this point forwards referred to as Gln^+7^) was detected in the Arabidopsis roots even after just 15 min of exposure to the labelled AA. The concentration of Gln^+7^ in the Arabidopsis roots was found to be constant over time (Fig. 1a). Consequently, the calculated uptake rates of Gln^+7^ decreased with increasing incubation time from 12.7 µmol h^−1^ g^−1^ DW after exposure for 15 min to 1.8 µmol h^−1^ g^−1^ DW after 120 min, a 7.1‐fold decrease.

### BSIA analysis of ^15^N and ^13^C

In contrast with the root tissue concentration of the intact Gln^+7^ molecule, the concentration of ^15^N increased significantly over time (Fig. 1b). However, like the LC‐MS data, the corresponding calculated uptake rates of ^15^N decreased with increasing incubation time: 25.9 µmol h^−1^ g^−1^ DW after exposure for 15 min and 8.8 µmol h^−1^ g^−1^ DW after exposure for 120 min, a 2.9‐fold decrease. The amount of ^13^C detected also increased significantly over time although not in proportion to the increase in ^15^N and, consequently, the ratio ^13^C‐labelled C : ^15^N‐labelled N decreased with time from 2.51 ± 0.008 after exposure for 15 min to 2.13 ± 0.01 after exposure for 120 min (Table 1). Given the 2.5 ratio for ^13^C : ^15^N in Gln^+7^, the amount of ^15^N found in the roots and because the experiment was carried out under axenic conditions, it was possible to calculate a theoretical ^13^C loss. Based on those calculations and because there had been uptake of intact Gln^+7^, we found that up to 15.2% ± 0.4% of the ^13^C in the root tissue was lost post‐uptake (Table 1).

**Table 1:** Arabidopsis root concentration of ^15^N and ^13^C after 15–120 min of exposure to U‐^15^N_2_, ^13^C_5_‐Gln solution, determined using bulk stable isotope analysis.

Method	Time (min)	^15^N (µmol g^−1^ DW)	^13^C (µmol g^−1^ DW)	Ratio (^13^C : ^15^N)	Estimated ^13^C loss (%)
BSIA	15	13.0 ± 0.5^a^	32.5 ± 1.4^a^	2.51 ± 0.008^a^	0
BSIA	30	18.4 ± 1.2^ab^	45.1 ± 2.9^ab^	2.46 ± 0.002^b^	1.8 ± 0.1
BSIA	45	22.8 ± 2.1^b^	53.6 ± 5.1^b^	2.35 ± 0.008^c^	6.1 ± 0.3
BSIA	60	25.0 ± 1.1^b^	56.6 ± 2.5^bc^	2.27 ± 0.007^d^	9.4 ± 0.3
BSIA	120	35.2 ± 3.4^c^	74.8 ± 7.6^c^	2.13 ± 0.010^e^	15.2 ± 0.4

Ratio of root ^13^C to ^15^N concentration and estimated ^13^C lost, given that ^15^N was taken up as U‐^15^N_2_, ^13^C_5_‐Gln. Values represent mean ± SE, *n* = 5. Different lowercase letters indicate significant differences between time points for each category (one‐way analysis of variance, Tukey's test).

### Quantitative comparison between BSIA and LC‐MS data

For the AA isotopologues, the LC‐MS data output did not distinguish between ^15^N and ^13^C labelling. To be able to compare the amounts of ^13^C and ^15^N labels that were incorporated into the AA pool on the basis of the BSIA data, the concentration of the individual isotopologues was multiplied by their masses compared with the monoisotopic form, resulting in the label concentration (e.g. the concentration of Gln^+7^ was multiplied by seven).

The amount of free Gln^+7^ detected using LC‐MS represented 48.7% of the ^15^N and ^13^C in the root material as measured using BSIA after exposure for 15 min and decreased to 21.8% after exposure for 120 min (Table 2). Making the same comparison for the sum excess label of all Gln isotopologues, 72.5% of the ^15^N and ^13^C detected using BSIA was found after exposure for 15 min and 43.4% after exposure for 120 min. Expanding the LC‐MS analysis to all analysed free AAs, eight AAs and NH_4_^+^ were found to carry detectable excess label compared with the unlabelled control (Supporting Information Fig. S1; Table S1). The sum of excess label found in those AA isotopologues and NH_4_^+^ represented 89.0% of the ^15^N and ^13^C, quantified using BSIA after exposure for 15 min and 58.7% after exposure for 120 min (Table 2).

**Table 2:** First two columns: the sum of ^15^N and ^13^C Arabidopsis root content obtained using bulk stable isotope analysis, and the sum of ^15^N and ^13^C found in the isotopologues of Gln, Asn, Glu, Asp, Ser, Gly, Ala and γ‐aminobutyric acid (GABA) obtained using liquid chromatography‐mass spectrometry (mean ± SE, *n* = 5).

Time (min)	Sum label (µmol ^15^N + ^13^C g^−1^ DW)		Recovery (%)		
	BSIA	LC‐MS	LC‐MS vs BSIA	Gln ^+7^ vs BSIA	Gln^tot label^ vs BSIA
15	45.5 ± 1.7	40.6 ± 2.0	89.0 ± 1.6	48.7 ± 1.1	72.5 ± 1.9
30	63.4 ± 3.6	51.8 ± 5.0	80.9 ± 3.2	37.2 ± 2.3	60.6 ± 3.1
45	76.4 ± 6.4	55.0 ± 5.7	71.3 ± 2.2	29.0 ± 1.4	51.8 ± 2.0
60	81.7 ± 3.2	54.7 ± 1.5	67.1 ± 1.1	25.9 ± 0.1	48.8 ± 0.5
120	110 ± 9.8	65.4 ± 7.7	58.7 ± 1.9	21.8 ± 1.3	43.4 ± 1.6

The three columns to the right show the recovery (%) of label in all AA isotopologues, Gln^+7^, and all Gln isotopologues in relation to that obtained using IRMS.

### Post‐uptake metabolism of AAs

**Fig. 2:**
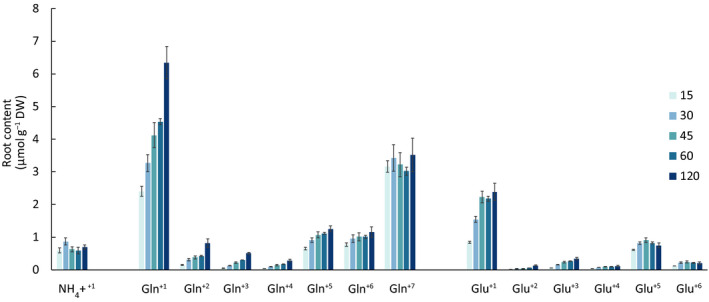
Isotopologue spectra of the two most ^13^C and ^15^N enriched amino acids and ammonium in Arabidopsis roots after 15–120 min exposure to U‐^15^N_2_, ^13^C_5_‐Gln solution, determined by LC‐qTOF. Mean ± SE, *n* = 5.

The concentrations of free AAs, including that of Gln, were mostly unaffected during the 120 min experiment (Table S1). However, the uptake and post‐uptake metabolism of dual‐labelled Gln changed the excess abundance of the ^+1^ or higher mass isotopologues of eight AAs. For Gln, as already stated, the concentration of Gln^+7^ was constant, but the composition of the other Gln isotopologues changed over time, with high enrichment of the ^+1^ isotopologue (Fig. 2; Table S1). Gln^+2^, Gln^+3^, Gln^+4^, Gln^+5^ and Gln^+6^ were also detected in the roots, with Gln^+5^ and Gln^+6^ being the more abundant (Fig. 2; Table S1). The second most abundant labelled AA was glutamate (Glu), with pronounced labelling in Glu^+1^, followed by Glu^+5^ and Glu^+6^. For the remaining AAs – aspartic acid (Asp), asparagine (Asn), GABA, alanine (Ala), serine (Ser) and glycine (Gly) – the label was mainly found in the ^+1^ isotopologue but for Asp and GABA, labelling of the ^+4^ isotopologue was also found.

To assess the whereabouts of label in the free AA pool, the isotopologue concentrations were multiplied by the number of labelled atoms (as described in the previous section). After exposure for 15 min, Gln isotopologues accounted for 81.5% of the ^15^N and ^13^C label in the pool of eight AAs carrying excess label (Gln^+7^ 54.7% and remaining Gln isotopologues 26.8%), the remaining 18.5% was mainly Glu (12.3%) and the sum fraction of GABA, Asp, Ala, Gly, Asn and NH_4_^+^ was 6.2% (Fig. S1). After exposure for 120 min, Gln isotopologues made up 74.1% of the ^15^N and ^13^C label in the pool of eight AAs. The fraction of Gln^+7^ decreased after 120 min exposure (37.7%) and the remaining Gln isotopologues increased to 36.4%. Glu increased to 14.0% after 120 min exposure and the sum fraction of the minor AAs increased to 11.9% (Fig. S1).

## Discussion

The transient nature of AAs makes quantification of their uptake in plants challenging. When applying dual‐labelled (^13^C, ^15^N) AAs as tracers for uptake quantification it still remains difficult to distinguish the parallel activities of AA metabolism of the intact AA molecule (post‐uptake metabolism) by the plant from pre‐uptake microbial cleavage (Warren, 2012; Czaban *et al*., 2016). In both cases, the critical factor is time.

Historically, the most frequently used method was BSIA, but this has been criticised for overestimating uptake rates (Sauheitl *et al*., 2009; Czaban *et al*., 2016; Dion *et al*., 2018). The increasingly popular compound‐specific isotope methods are associated with a risk of underestimating uptake due to plant post‐uptake metabolism. We therefore acknowledged the urgent need to unravel the influence of time on quantification by these methods.

To investigate the influence of post‐uptake metabolism on the quantification of uptake of the intact AA molecule, we applied U‐^13^C_5_‐^15^N_2_‐l‐Gln to axenically grown *A. thaliana* roots. Based on previous studies, in which post‐uptake metabolism of Gln was found to be rapid (Warren, 2012; Ganeteg *et al*., 2017), we examined a time frame of 15–120 min of exposure. From the first time point to the end, the root content of intact Gln^+7^ was constant, but the ^15^N and ^13^C content increased. Per unit of time, this led to very different calculated uptake rates.

We cannot rule out that the concentration of root‐available Gln decreased over time in the agar‐based experimental system, but the increase of ^15^N and ^13^C over the duration of the experiment underlined the fact that there was continuous uptake. The absolute uptake rate values might therefore have been affected but were regardless within the range of previously published data (Svennerstam *et al*., 2011). Although absolute uptake rate values may have been influenced by the concentration of root‐available Gln, this did not *per se* affect the comparison of BSIA and LC‐MS isotopic label quantification presented in this study. After as few as 15 min, analysed Gln^+7^ corresponded to only 48% of total ^13^C and ^15^N content and even less (22%) after 120 min. This underlines the fact that compound‐specific methods give lower estimates of AA uptake than BSIA; this is in line with previous studies using CSIA and LC‐MS (Sauheitl *et al*., 2009; Warren, 2012) but here in the absence of soil. The explanation for this discrepancy under axenic conditions is the post‐uptake metabolism of the labelled Gln that has been supplied.

The constant level of Gln^+7^ in the Arabidopsis roots, accompanied by the predominant enrichment of AA^+1^ isotopologues, as well as the enrichment of AA^+4^ and more enriched AA isotopologues, clearly demonstrated that the Gln that is taken up goes directly into the internal Gln pool and the metabolism of the plant. Ganeteg *et al*. (2017) observed the same pattern for Gln, in which the ratio of Gln^+7^ to ^15^N taken up declined from 106% to 25% for 15–60 min exposure, with simultaneous production of isotopologues of Gln. This is not surprising given the central role of Gln in AA metabolism, being a nitrogen donor during the synthesis of many AAs. The observed enrichment of the AA^+1^ isotopologues is likely to be the result of ^15^N transfer from Gln^+7^. As a consequence of deamination and deamidation of Gln^+7^ and the presence of unlabelled NH_4_^+^ (from the pre‐existing internal pool) as substrates, Gln/Glu^+5,+6^ isotopologues will also be produced. Therefore, the bulk of excess label was found in Gln and Glu, primarily as Gln/Glu^+1,+2,+5,+6^ isotopologues, suggesting that the GS‐GOGAT cycle is the main hub for Gln^+7^ post‐uptake metabolism. Only a relatively small amount of label was found in Asp, GABA, Ser, Gly, Ala and Asn; this was expected given their less central role in AA metabolism compared with Gln. This underlined that AAs are integrated into metabolism after uptake (Schmidt & Stewart, 1999) and that in using compound‐specific methods it is important to choose a time frame that captures the transient nature of AA, if it is to be used for quantitative purposes rather than qualitative measures.

Time is also crucial for AA uptake quantification with BSIA, although at a slightly different time scale than for compound‐specific methods. The present study highlights the fact that the effect of AA metabolism is not only the movement of isotope label from the molecule taken up to other isotopologues, but also carbon loss due to CO_2_ production (Persson & Näsholm, 2001; Warren, 2012; Ganeteg *et al*., 2017). Under the axenic conditions in the present study the ^13^C : ^15^N ratio declined from 2.51 at 15 min to 2.13 at 120 min, indicating that up to 15% of the Gln carbon was lost, post uptake, during this time. This corresponded approximately to the amount of label found in the different AA isotopologues. The most obvious candidate pathway for carbon loss is tricarboxylic acid cycle (TCA)‐cycle decarboxylation of 2‐oxoglutarate, the carbon backbone of Gln and Glu. Although no direct measurements of 2‐oxoglutarate isotopologues were made in this experiment, the enrichment of Asp^+4^ suggests that Gln^+7^‐derived 2‐oxoglutarate has entered the TCA cycle (the hypothesis is that Asp^+4^ is the product of oxaloacetate^+4^, a TCA‐cycle downstream metabolite from 2‐oxoglutarate^+5^). Another candidate for the loss of ^13^C is the decarboxylation of Glu to form GABA, as suggested by the presence of GABA^+4^. Because so many processes occur simultaneously, tracing is complex. However, by evaluating the time scale of pre‐uptake and post‐uptake events, the individual importance could be evaluated. Our study indicated that post‐uptake metabolism would not affect BSIA estimates of AA uptake during the first 15 min of exposure, whereas compound‐specific analysis would only quantify approximately half of the amount intact molecule taken up. Nevertheless, within 60 min there was less than 10% effect of post‐uptake metabolism on the BSIA results. As with post‐uptake metabolism, pre‐uptake metabolism of individual AA molecules in soil starts instantaneously and will therefore affect AA availability (Enggrob *et al*., 2019; Hill & Jones, 2019). Carbon loss during pre‐uptake metabolism is within the same order of magnitude during the first 60 min (pre‐uptake: 12% of added AA carbon to the soil), but the presence of the added intact molecule in the soil will be gone in minutes (Hill & Jones, 2019). In soil, however, the pool of AA will be replenished continuously by depolymerisation of peptides and proteins, which in a natural setting, in soil, increase plant uptake of N in organic form (Enggrob *et al*., 2019). It is important to keep in mind that these time frames are likely to be affected by temperature, AA concentrations, plant species, soil type, etc.

The difference between compound‐specific AA uptake and BSIA presented in previous research has a large range: 1.6%–106% (Sauheitl *et al*., 2009; Warren, 2012; Czaban *et al*., 2016; Ganeteg *et al*., 2017; Dion *et al*., 2018). There is great variation in the concentrations used in different studies and the largest difference between BSIA and the compound‐specific analysis has been reported in studies using the highest concentrations (Warren, 2012; Dion *et al*., 2018). Amino acid uptake rates follow Michaelis–Menten kinetics, that is increases with increased concentration (Soldal & Nissen, 1978; Svennerstam *et al*., 2011). In addition, uptake rates of different AAs vary (Forsum *et al*., 2008; Svennerstam *et al*., 2011), as do the rates at which they are metabolised. Amino acids do have different chemical properties and biological roles and occurrences. In this context, there seems to be a difference between the AAs that are central to AA metabolism and those that are metabolically more peripheral (Schmidt & Stewart, 1999; Warren, 2012), with the former (e.g. Gln, Asn) having a faster turnover (Warren, 2012; Czaban *et al*., 2016; Ganeteg *et al*., 2017; Dion *et al*., 2018) than the latter (e g Gly) (Warren, 2012). In experiments in which peripheral AAs have been used, no metabolic tracers were detected in the other AA pools (Sauheitl *et al*., 2009). The analysis in Sauheitl *et al*. (2009) differed slightly from that in other studies, analysing the total AA pool in the plant material (including AAs incorporated into proteins). An experimental duration of 24 h did increase the likelihood of labels being incorporated into proteins. However, by hydrolysing the samples used for protein AA analysis, AAs such as Gln and Asn are degraded, eliminating their possible use as tracers. More importantly, when including AAs residing in proteins in the analysis and, given that the amount of protein‐bound AA is *c*. 100–1000 times greater than that of the free AA pool, any AA tracer will be heavily diluted (Hildebrandt *et al*., 2015). This may affect the detection of label enrichment in the samples, especially for ^13^C, which is naturally more abundant than ^15^N (NA 1.1% and 0.4%). The analysis of Gln^+7^ (LC‐MS) circumvents potential dilution effects, as the naturally occurring proportion of this molecule is undetectable. In the present study, 65% of the ^15^N and ^13^C quantified by BSIA was detected by LC‐MS after 120 min. When accounting for potential carbon loss, presumably through respiration, up to 80% of the label was detected in the free AA pool. Therefore, for experiments with a duration of up to 120 min, protein synthesis seems to play a minor role. Therefore, for short‐term experiments, and if only the free AA pool is being extracted, the signal‐to‐noise ratio may be greatly reduced.

Given the post‐uptake metabolism observed in this experiment, it can be argued that:

(**1**) At least in the context of Gln, post‐uptake metabolism can be responsible for substantial losses of carbon, suggesting that microbial decomposition before plant uptake is not the only process contribution to ^13^C loss when using ^13^C–^15^N dual‐labelled AAs to determine AA uptake in an ecological setting. It is, however, plausible that other AAs, with a less central role in plant metabolism would not be metabolised as quickly as Gln.

The same principle also applies to root AA export, as shown by Warren (2012), who found that labelled Gln could be traced to the shoot, whereas labelled Gly could not.

(**2**) Post‐uptake metabolism complicates the interpretation of BSIA data, but in the context of compound‐specific LC‐MS, post‐uptake metabolism could be regarded as something positive.

If only taking intact AA uptake into account, for example Gln^+7^ in this experiment, it is possible that the Gln^+7^ detected was at least partly present in the apoplast. Therefore, for this axenic labelling experiment, the coexistence of Gln^+1^, Gln^+2^, Gln^+5^, Gln^+6^ and Gln^+7^ isotopologues shows that post‐uptake metabolism has occurred, representing convincing evidence that uptake over the root plasma membrane had taken place. However, in an ecological setting it could be argued that the plant roots had acquired these isotopologues after pre‐uptake decomposition by microorganisms.

Probably the most important factor that will influence the similarity of results produced by the two analytical methods and the accuracy of the quantification, as already mentioned above, is time. As stated many times before, this is because of the fast turnover of AAs in the soil (Rousk & Jones, 2010; Hill & Jones, 2019). However, time is equally important in terms of post‐uptake plant metabolism (Warren, 2012; Ganeteg *et al*., 2017), as emphasised by the results of this study. In the present study, AA uptake was measured in the absence of microbes and soil. Despite this, the amount of Gln^+7^ in relation to ^13^C and ^15^N in the plant decreased over time. This, in parallel with the production of Gln isotopologues and isotopologues of other AAs, showed that this decrease was due to post‐uptake metabolism.

### Conclusion

Studies on AA uptake quantification cover experimental periods ranging from minutes to hours to days and months. The results presented here suggest that experimental duration has a significant effect when determining uptake rates and that compound‐specific analysis should primarily be used in experiments with a time frame of minutes rather than hours or days. The loss of carbon after exposure for 120 min suggested that it is not only pre‐uptake metabolism by microbes that accounts for the ^15^N : ^13^C ratio mismatch reported in many ecological studies, but also plant respiration of ^13^C. Here we highlight the effects of plant metabolism on interpretation of results from differing analytical methods. We stress the importance, for all methods, of short sampling times to obtain accurate measures of plant uptake of intact AAs.

## Author contributions

All work was conceived jointly by HS and SJ; the experimental design and methodology, performance of the experiments, chemical and data analysis and interpretation as well as the writing of the manuscript.

## Supporting information


**Fig. S1** Distribution of label in amino acids and ammonium in Arabidopsis roots.
**Table S1** Arabidopsis root NH_4_^+^ and amino acid concentrations and individual isotopologue concentrations.
Please note: Wiley Blackwell are not responsible for the content or functionality of any Supporting Information supplied by the authors. Any queries (other than missing material) should be directed to the *New Phytologist* Central Office.

## Data Availability

The data that support the findings of this study are available from the corresponding author upon reasonable request.
