# Supplementary material for: Timing is everything – obtaining accurate measures of plant uptake of amino acids
Source: New Phytol. 2022 Feb 2;234(1):311–8. doi: 10.1111/nph.17964 (PMC9303729; doi:10.1111/nph.17964)
Supplement: Supplementary file 1 — Fig. S1 Distribution of label in amino acids and ammonium in Arabidopsis roots. Table S1 Arabidopsis root NH4 + and amino acid concentrations and individual isotopologue concentrations. Please note: Wiley Blackwell are not responsible for the content or functionality of any Supporting Information supplied by the authors. Any queries (other than missing material) should be directed to the New Phytologist Central Office. [file NPH-234-311-s001.pdf]

***New Phytologist* Supporting Information**

Article title: Timing is everything – obtaining accurate measures of plant uptake of amino acids
Authors: Henrik Svennerstam and Sandra Jämtgård
Article acceptance date: 22 December 2022

The following Supporting Information is available for this article:

Fig. S1 Distribution of label in amino acids and ammonium in Arabidopsis roots.

Table S1 *Arabidopsis* root NH_4^+ and amino acid concentrations (in bold) and individual isotopologue concentrations.

Fig. S1 Distribution of label in amino acids and ammonium in *Arabidopsis* roots after a) 15 min and b) 120 min exposure to U-$^{15}N_2$, $^{13}C_5$-Gln solution, determined by LC-qTOF. Values represent mean, n=5.

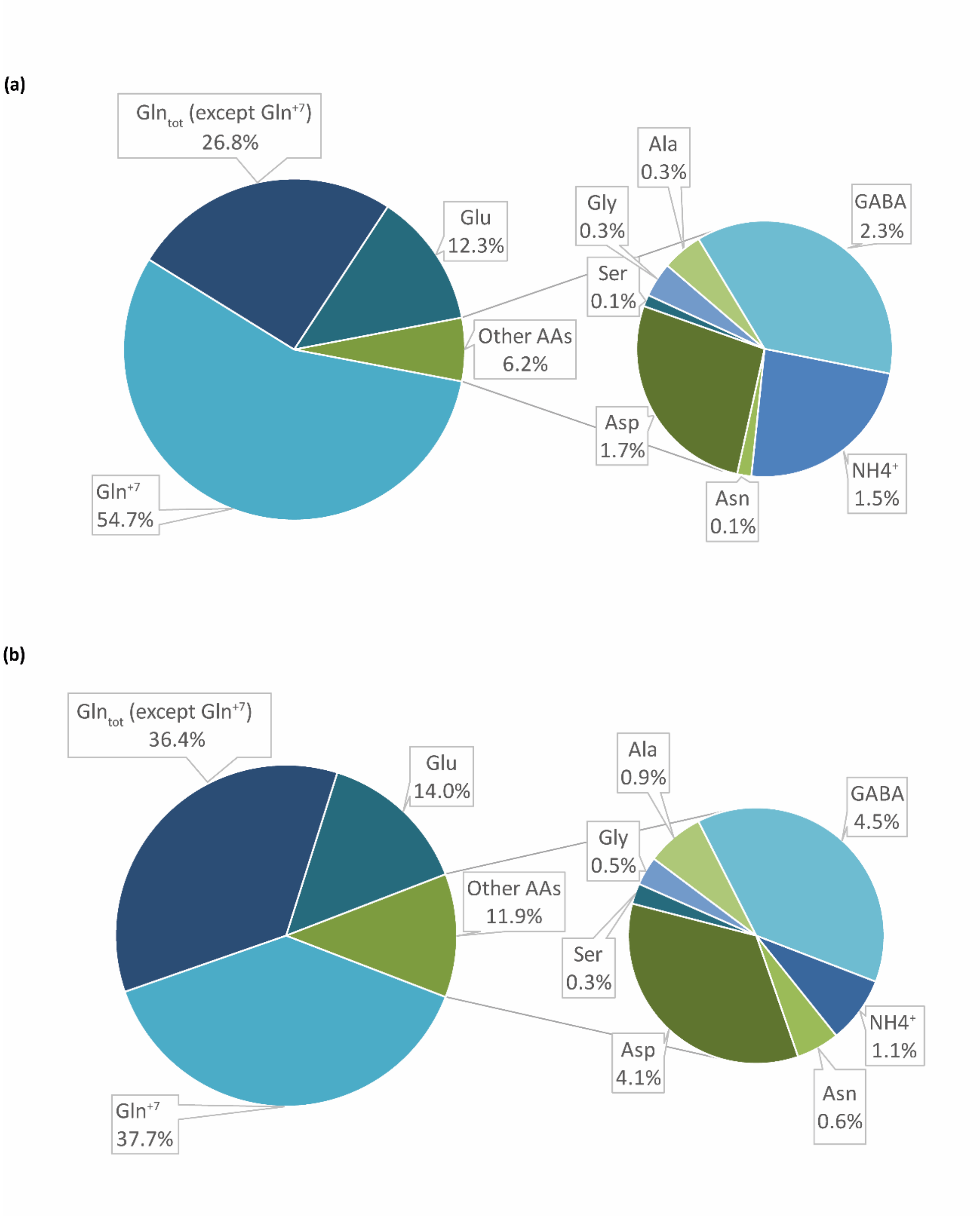

Table S1 *Arabidopsis* root NH_4^+ and amino acid concentrations (in bold) and individual isotopologue concentrations (µmol g^{-1} dw). Values represent mean ± SE, n=5. Different lower-case letters indicate differences between time points within each category (One-way ANOVA, Tukey's test).

	**Exposure time (min)**					
	**0**	**15**	**30**	**45**	**60**	**120**
**NH_4^+ tot**	**18.09 ± 0.58^{a}**	**14.57 ± 0.60^{ab}**	**16.48 ± 0.56^{ab}**	**13.83 ± 0.78^{ab}**	**13.10 ± 2.06^{b}**	**13.70 ± 0.36^{ab}**
**NH4^{+0}**		13.97 ± 0.53	15.61 ± 0.51	13.20 ± 0.72	12.51 ± 1.97	13.01 ± 0.34
**NH4^{+1}**		0.59 ± 0.07	0.87 ± 0.1	0.63 ± 0.08	0.59 ± 0.1	0.69 ± 0.07
**GLN ^{tot}**	**32.49 ± 1.35^{a}**	**33.03 ± 1.06^{a}**	**32.95 ± 1.75^{a}**	**34.15 ± 1.65^{a}**	**35.41 ± 1.28^{a}**	**37.23 ± 1.7^{a}**
**GLN^{+0}**		25.98 ± 0.66	24.04 ± 1.08	24.16 ± 0.64	25.01 ± 1.27	23.56 ± 0.90
**GLN^{+1}**		2.40 ± 0.14	3.27 ± 0.26	4.12 ± 0.39	4.54 ± 0.1	6.35 ± 0.49
**GLN^{+2}**		0.15 ± 0.01	0.32 ± 0.04	0.38 ± 0.05	0.42 ± 0.02	0.81 ± 0.13
**GLN^{+3}**		0.05 ± 0.00	0.13 ± 0.01	0.23 ± 0.02	0.30 ± 0.01	0.50 ± 0.04
**GLN^{+4}**		0.04 ± 0.00	0.10 ± 0.01	0.15 ± 0.02	0.17 ± 0.01	0.28 ± 0.04
**GLN^{+5}**		0.65 ± 0.03	0.91 ± 0.07	1.07 ± 0.1	1.11 ± 0.03	1.25 ± 0.09
**GLN^{+6}**		0.77 ± 0.05	0.96 ± 0.11	1.01 ± 0.12	1.01 ± 0.05	1.15 ± 0.16
**GLN^{+7}**		3.17 ± 0.18	3.43 ± 0.40	3.22 ± 0.37	3.02 ± 0.12	3.52 ± 0.52
**GLU ^{tot}**	**18.09 ± 0.6^{a}**	**15.86 ± 0.11^{a}**	**16.81 ± 0.53^{a}**	**17.47 ± 0.39^{a}**	**17.03 ± 0.84^{a}**	**15.90 ± 0.93^{a}**
**GLU^{+0}**		14.18 ± 0.13	13.95 ± 0.52	13.72 ± 0.12	13.40 ± 0.75	11.97 ± 0.65
**GLU^{+1}**		0.84 ± 0.03	1.55 ± 0.09	2.22 ± 0.18	2.18 ± 0.08	2.39 ± 0.26
**GLU^{+2}**		0.01 ± 0.00	0.03 ± 0.01	0.03 ± 0.00	0.06 ± 0.00	0.13 ± 0.01
**GLU^{+3}**		0.05 ± 0.00	0.16 ± 0.01	0.24 ± 0.02	0.26 ± 0.01	0.34 ± 0.03
**GLU^{+4}**		0.04 ± 0.00	0.07 ± 0.00	0.10 ± 0.01	0.09 ± 0.00	0.11 ± 0.02
**GLU^{+5}**		0.61 ± 0.01	0.82 ± 0.04	0.91 ± 0.07	0.82 ± 0.03	0.75 ± 0.07
**GLU^{+6}**		0.12 ± 0.01	0.22 ± 0.02	0.24 ± 0.03	0.22 ± 0.01	0.21 ± 0.04
**ASP ^{tot}**	**7.60 ± 0.63^{a}**	**7.10 ± 0.09^{a}**	**8.59 ± 0.32^{a}**	**8.45 ± 0.40^{a}**	**8.53 ± 0.37^{a}**	**7.93 ± 0.39^{a}**
**ASP^{+0}**		6.68 ± 0.08	7.59 ± 0.26	7.13 ± 0.25	7.18 ± 0.36	6.37 ± 0.28
**ASP^{+1}**		0.33 ± 0.02	0.72 ± 0.05	0.97 ± 0.11	0.98 ± 0.02	1.11 ± 0.12
**ASP^{+2}**		0.01 ± 0.00	0.04 ± 0.00	0.07 ± 0.01	0.08 ± 0.00	0.13 ± 0.01
**ASP^{+3}**		0.00 ± 0.00	0.02 ± 0.00	0.03 ± 0.00	0.03 ± 0.00	0.04 ± 0.01
**ASP^{+4}**		0.07 ± 0.00	0.18 ± 0.01	0.21 ± 0.02	0.21 ± 0.01	0.22 ± 0.02
**ASP^{+5}**		0.01 ± 0.00	0.03 ± 0.00	0.05 ± 0.01	0.05 ± 0.00	0.05 ± 0.01
**GABA ^{tot}**	**8.71 ± 0.54^{a}**	**6.76 ± 0.21^{b}**	**6.85 ± 0.20^{b}**	**6.39 ± 0.08^{b}**	**7.20 ± 0.27^{b}**	**7.51 ± 0.12^{ab}**
**GABA^{+0}**		6.37 ± 0.19	6.09 ± 0.15	5.55 ± 0.12	6.13 ± 0.26	6.01 ± 0.15
**GABA^{+1}**		0.21 ± 0.01	0.42 ± 0.03	0.5 ± 0.02	0.64 ± 0.02	0.93 ± 0.06
**GABA^{+2}**		0.01 ± 0.00	0.05 ± 0.00	0.06 ± 0.00	0.09 ± 0.00	0.15 ± 0.01
**GABA^{+3}**		0.01 ± 0.00	0.02 ± 0.00	0.02 ± 0.00	0.03 ± 0.00	0.04 ± 0.01
**GABA^{+4}**		0.13 ± 0.00	0.22 ± 0.01	0.22 ± 0.01	0.25 ± 0.01	0.30 ± 0.02
**GABA^{+5}**		0.03 ± 0.00	0.05 ± 0.01	0.05 ± 0.01	0.06 ± 0.00	0.07 ± 0.01
**ALA ^{tot}**	**5.08 ± 0.7^{a}**	**3.73 ± 0.05^{a}**	**4.38 ± 0.72^{a}**	**3.35 ± 0.15^{a}**	**3.51 ± 0.15^{a}**	**3.68 ± 0.12^{a}**
**ALA^{+0}**		3.61 ± 0.05	4.12 ± 0.68	3.07 ± 0.12	3.18 ± 0.15	3.18 ± 0.09
**ALA^{+1}**		0.1 ± 0.00	0.24 ± 0.04	0.25 ± 0.03	0.29 ± 0.01	0.45 ± 0.04
**GLY ^{tot}**	**10.16 ± 1.4^{a}**	**8.65 ± 0.09^{a}**	**10.16 ± 0.92^{a}**	**8.05 ± 0.18^{a}**	**8.72 ± 0.31^{a}**	**8.87 ± 0.24^{a}**
**GLY^{+0}**		8.54 ± 0.08	9.98 ± 0.91	7.86 ± 0.16	8.47 ± 0.32	8.57 ± 0.21
**GLY^{+1}**		0.11 ± 0.01	0.19 ± 0.02	0.19 ± 0.02	0.25 ± 0.02	0.31 ± 0.03
**SER ^{tot}**	**7.54 ± 2.35^{a}**	**4.79 ± 0.09^{a}**	**6.87 ± 1.80^{a}**	**4.28 ± 0.08^{a}**	**4.3 ± 0.20^{a}**	**4.62 ± 0.17^{a}**
**SER^{+0}**		4.76 ± 0.09	6.77 ± 1.78	4.17 ± 0.07	4.16 ± 0.20	4.42 ± 0.16
**SER^{+1}**		0.04 ± 0.01	0.10 ± 0.02	0.11 ± 0.01	0.14 ± 0.00	0.20 ± 0.01
**ASN ^{tot}**	**4.43 ± 0.29^{a}**	**4.12 ± 0.06^{a}**	**4.58 ± 0.15^{a}**	**4.35 ± 0.14^{a}**	**4.45 ± 0.29^{a}**	**4.82 ± 0.20^{a}**
**ASN^{+0}**		4.08 ± 0.07	4.50 ± 0.14	4.23 ± 0.13	4.31 ± 0.29	4.52 ± 0.19
**ASN^{+1}**		0.04 ± 0.01	0.07 ± 0.01	0.09 ± 0.01	0.12 ± 0	0.23 ± 0.02
**ASN^{+2}**		0 ± 0	0.01 ± 0	0.02 ± 0	0.01 ± 0	0.04 ± 0.01
